# Is maternal negative affectivity related to psychosocial behavior of preterm and term-born toddlers through mother–child interaction?

**DOI:** 10.3389/fpsyg.2022.975124

**Published:** 2022-10-03

**Authors:** L. J. G. Krijnen, M. Verhoeven, A. L. van Baar

**Affiliations:** Child and Adolescent Studies, Utrecht University, Utrecht, Netherlands

**Keywords:** negative affectivity, mother–child interaction, moderate to late preterm, social emotional development, moderated mediation, structured task, internalizing and externalizing problems, psychosocial problems

## Abstract

**Introduction:**

Children born moderately to late preterm (MLP) are more prone to psychosocial difficulties than their term-born counterparts. Maternal negative affectivity (NA)–a relatively stable personality trait characterized by the tendency to experience negative thoughts, feelings and emotions–has been related to more psychosocial problems in their offspring, and to a lower quality of mother–child interactions. As MLP children seem more sensitive to their early caregiving environment, they might be more affected by maternal NA and interaction style than their term-born peers. The current study investigated whether maternal NA predicted child’s psychosocial outcomes through quality of mother–child interaction, and if these associations differed between MLP and term-born children.

**Methods:**

The sample consisted of 108 MLP and 92 term-born children and their mothers. At 18 months corrected age, maternal NA was measured using a self-report questionnaire and mother–child interaction was observed during two structured tasks. Five subscales of mother–child interaction were assessed: negative interaction, reciprocal engagement, emotional support, maternal stimulation and mother-led interaction. At 24 months corrected age, social–emotional difficulties, internalizing, and externalizing problems were assessed using mother-report.

**Results:**

For MLP children, maternal NA directly, positively, predicted social–emotional difficulties (*b* = 0.57) and internalizing problems (*b* = 0.45), but no mediation effect of mother–child interaction was found. For term-born children, no direct effect but a mediation effect of mother-led interaction was found. Higher levels of maternal NA predicted less mother-led interaction which in turn predicted more problems. Birth status did not moderate any of the relationships, showing that the differences in patterns of effects found within the MLP and term-born group did not reach statistical significance.

**Discussion:**

Maternal NA was found to be a risk factor for psychosocial outcomes in toddlers, either directly for MLP children or indirectly through mother-led interaction for term-born children. These findings suggest that the process through which maternal NA affects psychosocial outcomes may be different for MLP and term-born children. However, as the examined moderation effects of birth status did not reach statistical significance, more research using larger sample sizes is needed to study mother–child interaction in greater detail.

## Introduction

Approximately, 1 in 10 children is born preterm (i.e., gestational age of <37 weeks), of which 85% is considered moderate to late preterm (MLP; gestational age 32–37 weeks) (Chawanpaiboon et al., 2019). Compared to term-born children, MLP children are more prone to psychosocial difficulties, such as internalizing – e.g. anxious and depressed moods – and externalizing problems – e.g. attentional problems, aggression, and a lower self-control (Talge et al., 2010; Potijk et al., 2012). However, not every MLP child develops psychosocial difficulties, indicating that other factors play a role. Research has shown that the early caregiving environment – e.g. parenting behaviors, parental characteristics – forms an important contributor to the psychosocial development, with evidence that preterm children are more affected by this than their full-term counterparts (Gueron-Sela et al., 2015). Therefore, it is important to identify which early caregiving factors increase the risk of developing psychosocial difficulties in MLP children. Possibly, interventions for psychosocial difficulties in MLP children can be adjusted towards targeting such relevant risk factors.

For both MLP and term-born children, maternal depressive and anxiety symptoms have been studied extensively and have repeatedly been linked to more internalizing and externalizing problems in their offspring (Brennan et al., 2000; Barker et al., 2011; Goodman et al., 2011; Rogers et al., 2013). Premature infants however, including MLP infants, were found to be exceptionally hormonally sensitive to maternal depressive symptoms, as they showed higher cortisol levels compared to full-term children who were also at medical risk (Bugental et al., 2008). Furthermore, mothers with depression reported lower social abilities – e.g. ability to make friends, share with others, play independently – for preterm born toddlers but not for term-born toddlers (Silverstein et al., 2010). Additionally, high maternal emotional distress, as measured by anxiety, stress and depressive symptoms, was found to impact social competences of all children, but especially in preterm children (Gueron-Sela et al., 2015). This indicates that the emotional state of mothers is particularly important for preterm infants.

Recent literature showed that especially the *stable* trait portion of maternal anxiety and depressive symptoms – more than the transient elevated anxiety and depression symptomatology–is predictive for child’s psychosocial outcomes at age 2 (Prenoveau et al., 2017) and also at age 12.5 (Missler et al., 2021). These effects were also found for subclinical levels of maternal depression and anxiety, highlighting the need to shift the research focus from clinical depressive and anxiety diagnosis to subclinical, stable traits that underly these disorders (Kingston et al., 2018; Missler et al., 2021). A relatively stable personality trait that is described as an underlying common risk factor for depressive and anxiety disorders is negative affectivity (NA) (Watson and Clark, 1984; Watson et al., 2011; Stanton and Watson, 2014). NA is characterized by the tendency to experience negative thoughts, feelings and emotions across time and regardless of situations (Watson and Clark, 1984; Denollet, 2013). High NA individuals tend to take a gloomy view of things and are prone to feelings of dysphoria, anxiety and irritability even in the absence of an objective stressful event (Watson and Clark, 1984; Denollet, 2013). Maternal NA has not widely been studied yet in relation to MLP versus term-born psychosocial outcomes, which is why the current study will investigate the role of maternal NA further. Due to the lack of research on this topic, we will mostly discuss previous literature about maternal depressive and anxiety symptoms as these concepts are close to NA.

There is evidence that mothers with depression behave differently towards their child, resulting in a lower quality of mother–child interaction, which in turn may lead to more psychosocial difficulties (Dubois-Comtois et al., 2013; Villodas et al., 2015). Therefore, quality of mother–child interaction may be a mediating factor between maternal NA and child’s psychosocial outcomes. For term-born children, previous research showed that mothers with depressive symptoms displayed more mother–child aggression – e.g. aggressive interactions, harsh disciplining – during early childhood, which in turn predicted more externalizing behavior during middle childhood (Villodas et al., 2015). Furthermore, maternal psychosocial distress predicted a lower quality of mother–child interaction – i.e. characterized by low levels of reciprocated, open and balanced communication – which in turn predicted more child’s reported internalizing and externalizing problems at age 8.5, showing a mediation effect of mother–child interaction (Dubois-Comtois et al., 2013). Another study in children aged 8–12 years with externalizing problems showed that maternal depressive symptoms predicted lower maternal warmth during mother–child interaction and more mother-reported internalizing and externalizing problems in the child. However, maternal warmth did not mediate the relation between maternal depressive symptoms and the child’s internalizing and externalizing behavior (van Doorn et al., 2016). These studies indicate that higher levels of depressive symptoms or psychosocial distress in mothers may lead to a lower quality of mother–child interaction, which in turn affects children’s psychosocial outcomes. However, previous findings are inconsistent regarding the mediating role of mother–child interaction.

The question is whether preterm children are more affected by a lower quality of mother–child interaction than full-term children. It seems that preterm children may be more sensitive to their early caregiving environment. To illustrate, if mothers were consistently responsive to their child in the first 4 years of life, cognitive growth was faster for all children, but this effect was stronger for preterm children than for term-born children (Landry et al., 2001). Additionally, an intervention targeting maternal responsiveness led to better social and emotional skills, again especially in the preterm group (Landry et al., 2006). A study by Gueron-Sela et al. (2015) also found evidence that premature born children are more affected by their early caregiving environment than term-born children. They found that in families with high maternal stress and a lower quality of parent–child interaction at 6 months, social competences at 12 months were lower for preterm children than full-term children. Conversely, when maternal stress was low and the quality of interaction was high, preterm children outperformed their full-term peers in terms of social competences (Gueron-Sela et al., 2015). This indicates that prematurely born children might be more affected by their early caregiving environment than term-born children.

**Figure 1:**
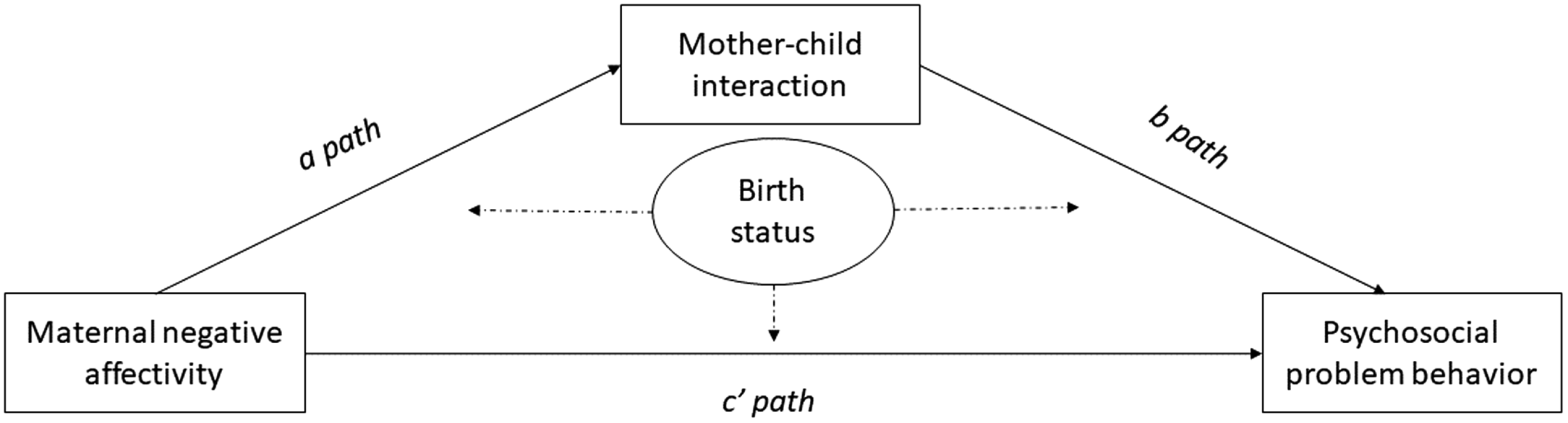
The proposed model. Dashed lines indicate moderation effects.

The current study will investigate whether the relation between maternal NA and toddler’s psychosocial functioning (i.e., social–emotional difficulties, internalizing, and externalizing problem behavior) is mediated by the quality of mother–child interaction, and whether these relationships are different in MLP versus term-born children (i.e., birth status) (see Figure 1). It is hypothesized that higher levels of NA in the mother will predict more psychosocial difficulties in their offspring. It is expected that this relationship is stronger for preterm children as these children seem more sensitive to depressive symptoms of the mother – a concept that is related to NA (Bugental et al., 2008) (See c’ path Figure 1). It is expected that this link between maternal NA and psychosocial outcomes is mediated by the quality of mother–child interaction, with higher levels of maternal NA being related to a lower quality of mother–child interaction (See *a* path Figure 1) which predicts more psychosocial difficulties in the child (See *b* path Figure 1). This mediation effect is expected to be stronger in preterm children than in term-born children as premature children may be more sensitive to both maternal NA and the quality of mother–child interaction.

## Materials and methods

### Participants and procedure

This study is part of a larger longitudinal study called the Study on Attention of Preterm children (STAP) project, in which MLP and term-born children took part, all born between March 2010 and April 2011. Data was collected from March 2011 to March 2013. All children were recruited in nine hospitals around Utrecht, Netherlands. Pediatricians and midwives asked parents to participate when the child was 10 months old. Exclusion criteria were severe congenital malformations, dysmaturity, multiple births, admission to a tertiary Neonatal Intensive Care Unit (NICU), maternal antenatal substance abuse or chronic antenatal use of psychiatric drugs. The STAP project was approved by the Utrecht Medical Center Ethics Committee (identification code NL34143.041.10) and both parents provided written informed consent.

The initial sample consisted of 226 participants. Assessments took place when the child was 18 months and 24 months of corrected age for prematurity. At 18 months, mothers were asked to fill out a questionnaire to measure NA, and mother–child interaction was assessed. At 24 months, the mother filled out the measurements regarding the child’s psychosocial functioning. Participants that had no data on maternal NA, mother–child interaction or none of the psychosocial outcomes were removed from the dataset, and one participant that exceeded the age range for filling out the Ages and Stages Questionnaire 24 month version (i.e., 30 months). The final sample consisted of 200 children, of which 108 MLP and 92 term-born. See Table 1 for the participant’s characteristics.

**Table 1:** Participant characteristics per group of birth status.

	MLP (*n* = 108)	Term (*n* = 92)
Gender		
Male, *n* (%)	63 (58.33%)	41 (44.57%)
Female, *n* (%)	45 (41.67%)	51 (55.43%)
Corrected age in months, wave 1		
Mean (SD)	17.22 (0.44)	17.32 (0.47)
Range	17–19	17–18
Corrected age in months, wave 2		
Mean (SD)	23.32 (0.54)	23.59 (0.63)
Range	23–25	23–26
Ethnicity, *n* (% Dutch)	104 (96.30%)	88 (96.30%)
Gestational age		
Mean (SD)	34.69 (1.34)^***^	39.47 (0.99)
32 weeks, *n* (%)	11 (10.19%)	
33 weeks, *n* (%)	11 (10.19%)	
34 weeks, *n* (%)	19 (17.59%)	
35 weeks, *n* (%)	27 (25.00%)	
36 weeks, *n* (%)	40 (37.04%)	
37 weeks, *n* (%)		4 (4.35%)
38 weeks, *n* (%)		9 (9.78%)
39 weeks, *n* (%)		31 (33.70%)
40 weeks, *n* (%)		36 (39.13%)
41 weeks, *n* (%)		12 (13.04%)
Birth weight in grams		
Mean (SD)	2584.77***	3575.44
Range	1,420–3,850	2,795–5,330
Education level mother^a^		
Low, *n* (%)	7 (6.48%)	2 (2.17%)
Medium, *n* (%)	36 (33.33%)^***^	10 (10.87%)
High, *n* (%)	65 (60.19%)^***^	80 (86.96%)

MLP, moderate to late prematurely born children; SD, standard deviation. To test for groups differences, *t*-tests and Fisher’s exact tests were used.

a Low: no education, special education, elementary school, lower secondary education; Medium: secondary or vocational education; High: college, university or higher.

* *p* < 0.05;

** *p* < 0.01;

*** *p* < 0.001.

#### Negative affectivity (18 months)

Maternal NA was measured using the Type D Scale-14 (DS14) (Denollet, 2005). The DS14 measures two scales: Negative Affectivity (7 items) and Social Inhibition (7 items) of which we only used the former scale to assess NA. The NA scale covers dysphoria (e.g., “I often feel unhappy”), worry (e.g., “I often find myself worrying about something”) and irritability (e.g., “I am often irritated”) and items are answered on a scale from 0 = “false” to 4 = “true.” Sum scores were calculated by adding the seven NA items, leading to sum scores within the range of 0 to 28, with higher scores indicating more NA. The DS14 has shown to be a psychometrically sound instrument (Denollet, 2005), also cross-culturally (Kupper et al., 2013). The NA scale was previously shown to be internally consistent (α = 0.88) (Denollet, 2005). The internal consistency was α = 0.87 in the current study.

#### Mother–child interaction (18 months)

Mother–child interaction was observed during structured tasks, as such tasks are known to elicit more differential mother–child interactions than free-play settings in which interactions are mainly positive and less varied (Ginsburg et al., 2006). Mothers were asked to perform two structured tasks with their child: (1) reading a book (5 min), (2) making a puzzle together (5 min). Interactions were videotaped and trained raters coded the behaviors using the Coding Interactive Behavior Manual (CIB; Feldman, 1998). The CIB is a global rating scheme for children aged 2 to 36 months and assesses the frequency of certain behaviors (e.g., joint attention, intrusiveness, positive affect). These behaviors are measured from the child’s perspective (16 items), from the mothers’ perspective (21 items) and their dyadic interaction (5 items). All items were scored by a trained assessor on a 5-point Likert scale ranging from 1 (little) to 5 (much). Inter-rater reliability was calculated based on 21% double coded videos, and was acceptable (ICC = 0.76).

There are no pre-distinguished subscales for the CIB and studies differ in which behaviors are grouped together to form subscales (Feldman et al., 2002; Feldman, 2010; Weisman et al., 2015). We therefore conducted an exploratory factor analysis to discover which subscales best represented the mother–child interaction characteristics in the current study. This led to the following 5 subscales: (1) Negative interaction, consisting of child’s positive affect (reversed), child’s negative emotionality, child’s labile affect, child’s avoidant behavior, dyadic constriction and dyadic tension (α = 0.89); (2) Reciprocal engagement, consisting of child’s joint attention, child’s on task persistence, child’s withdrawal (reversed), child’s compliance to parent, child’s initiation, dyadic reciprocity, dyadic affect-regulation, dyadic fluency (α = 0.85); (3) Maternal stimulation, consisting of parents’ elaborating, parents’ resourcefulness, parents’ on task persistence, parents’ limit setting (α = 0.81); (4) Emotional support consisting of parent’s acknowledgement, parents’ positive affect, parents’ negative affect (reversed), parents’ supportive presence, parents’ appropriate range of affect (α = 0.75); (5) Mother-led interaction, consisting of parent’s intrusiveness, child-led interaction (reversed), child’s affection towards parent, child’s reliance on parent for help (α = 0.68). Average scores were calculated by adding the items of the relevant behaviors per subscale and dividing it by the number of items. Scores on each subscales could range between 1 and 5, with higher scores indicating that the behaviors of the subscale are more characteristic for the mother–child dyad.

#### Social–emotional difficulties (24 months)

Social–emotional difficulties of the child was measured at 24 months corrected age using the Dutch translation of the Ages and Stages Questionnaire-Social Emotional (ASQ-SE; Squires et al., 2002). The ASQ-SE is a parent-report screening instrument that aims to detect children with social–emotional difficulties and delays by addressing seven behavioral dimensions: self-regulation, compliance, social-communication, adaptive functioning, autonomy, affect, and interaction with people. The 24 months age version, which can be used for children from 21 to 26 months of age, consists of 26 scored items which are answered with “most of the time” (0 points) “sometimes” (5 points) and “rarely/never” (10 points). For every question, the parent can express concerns regarding the child’s behavior, leading to an additional 5 points. A sum score is calculated by summing the points of the 26 items including the points of the concerns, in which a higher score relates to more social–emotional difficulties or delays. Scores could range between 0 and 390. When no more than 3 items were missing on the ASQ-SE, mean imputation was used as recommended (Squires et al., 2002). The ASQ-SE has shown good psychometric properties in the United States (Squires et al., 2002) and the Dutch translation has shown good specificity (De Wolff et al., 2013; Krijnen et al., 2021) and sufficient sensitivity (Krijnen et al., 2021) to slightly below the cut-off for sufficient sensitivity (i.e., 66%) (De Wolff et al., 2013). Internal consistency for the 24 months version has shown to be good, α = 0.80 (Squires et al., 2001). For the current sample, internal consistency was on the lower side, i.e., α = 0.45, though some studies still consider this sufficient (for an overview, see Taber, 2018). This lower internal consistency can be explained by the broad domain of social–emotional development that the ASQ-SE assesses, whereas an uni-dimensional structure is assumed for internal consistency measures. As the current study aims to get an indication of the social–emotional development of the child, the lower internal consistency in the current study is considered to not be of major concern.

#### Internalizing and externalizing problem behavior (24 months)

The Child Behavior Checklist 1½-5 (CBCL) (Achenbach and Rescorla, 2001) is a parent-report questionnaire measuring behavioral and emotional problems of children aged 1.5 to 5 years old over the past 2 months. For the current study, the two broad-band scales of internalizing and externalizing problem behavior were used. The internalizing scale (36 items) consists of the following 4 domains of behavioral problems: emotionally reactive (e.g., “disturbed by any change in routine”), anxious/depressed (e.g., “nervous, high-strung or tense”), somatic complaints (e.g., “headaches”), and withdrawn (e.g., “seems unresponsive to affection”). The externalizing scale (24 items) consists of 2 domains: attention problems (e.g., “cannot concentrate”) and aggressive behavior (e.g., “angry moods”). Questions are answered on a three-point scale ranging from 0 = “not true,” 1 = “somewhat or sometimes true” and 2 = “very true or often true.” Scores were calculated by summing the behaviors and standardized T scores were calculated, with higher scores indicating more problem behavior. T scores for the internalizing scale could range between 29 and 100, and for the externalizing scale between 28 and 100. The CBCL 1½-5 has shown good reliability and validity (Achenbach and Rescorla, 2001). The internal consistency of the two broad-band scales was good, with α = 0.89 for internalizing and α = 0.92 for the externalizing scales (Achenbach, 2011). In the current study, the internal consistency for the internalizing and externalizing scale was 0.75 and 0.88, respectively.

### Statistical analyses

R version 4.0.3 was used to analyze the data. Bivariate Pearson correlations among the variables were investigated and descriptive analyses were executed using independent *t*-tests to check for group differences between the MLP and term-born group. The PROCESS macro, written by Hayes (2017) was used to test the moderated mediation model. PROCESS uses ordinary least squares regression-based path analysis and estimates the moderating and mediating relationships simultaneously using observed variables or observed variable proxies (i.e., sum scores or averages of indicators) (Hayes et al., 2017). A single test procedure is used in which one statistic accounts for the indirect effect of X on Y through the mediator, enhancing its power and making it an increasingly used method in psychology research.

Maternal NA was added as the predictor variable and the five mother–child interactions were added as parallel mediators within one model (i.e., negative interaction, reciprocal engagement, maternal stimulation, emotional support and mother-led interaction), allowing the mediators to be correlated and estimating the parameters of each mediator while controlling for effects of the other mediators. Both the predictor and the mediators were mean centered to avoid multi-collinearity. Gender of the child (0 = male, 1 = female) and education level of the mother (low/medium/high, resulting in 3 dummy variables with low as the reference category) were added to the model as covariates, as both are known to be related to children’s psychosocial outcomes (Potijk et al., 2015; Stene-Larsen et al., 2016). Birth status was added as a dichotomous moderator (0 = term, 1 = MLP) and the moderated mediation model was run using PROCESS model 59. The moderated mediation model produces estimates of effects per level of the moderator, while testing whether these effects are significantly different between each other. The models were run three times, for every outcome measure separately (i.e., social–emotional development, internalizing problem behavior, externalizing problem behavior). Robust standard errors were used to protect against heteroscedasticity. Indirect effects were tested using a bootstrapping procedure with 10,000 iterations to protect against non-normality using seed 654321 for the random number generator. Indirect effects were considered significant when the 95% bootstrapped confidence interval excluded 0. Unstandardized regression coefficients were reported, following the recommendations of Hayes stating that these are easier to interpret as the unstandardized metric directly maps onto the scale of the variables (Hayes, 2017).

## Results

### Descriptives

See Table 2 for the scores on maternal NA, mother–child interaction and psychosocial behavior per group. MLP children scored significantly higher on internalizing problems than term-born children [*t*(193.37) = −3.02, *p* = 0.002]. No differences were found on social–emotional difficulties [*t*(189.86) = −1.56, *p* = 0.12] and externalizing problems [*t*(181.49) = −1.77, *p* = 0.08]. Concerning the mother–child interaction behaviors, differences were found for reciprocal engagement, which was significantly lower in the MLP group [*t*(197.81) = 2.46, *p* = 0.01]. Levels of maternal NA were not different between the groups [*t*(197.56) = −0.09, *p* = 0.92].

**Table 2:** Descriptive statistics.

	MLP (*n* = 108)	Term (*n* = 92)
Social–emotional difficulties^a^		
Mean (SD)	18.17 (11.99)	15.45 (12.50)
Range	0–50	0–65
Internalizing problems^b^		
Mean (SD)	44.76 (8.85)^**^	41.10 (8.15)
Range	29–67	29–58
Externalizing problems^b^		
Mean (SD)	48.87 (7.96)	46.73 (8.80)
Range	32–71	28–64
Mother-led interaction		
Mean (SD)	2.53 (0.85)	2.66 (0.83)
Range	1.00–4.50	1.00–4.25
Maternal stimulation		
Mean (SD)	3.71 (0.84)	3.89 (0.78)
Range	1.50–5.00	2.00–5.00
Reciprocal engagement		
Mean (SD)	3.61 (0.68)^*^	3.83 (0.60)
Range	1.75–5.00	2.38–4.88
Negative interaction		
Mean (SD)	1.31 (0.53)	1.28 (0.50)
Range	1.00–3.50	1.00–3.83
Emotional support		
Mean (SD)	4.78 (0.39)	4.82 (0.40)
Range	3.00–5.00	3.20–5.00
Maternal negative affect		
Mean (SD)	6.42 (5.17)	6.35 (4.61)
Range	0–21	0–22

MLP: moderate to late preterm born children; SD, standard deviation. To test for groups differences, *t*-tests were used.

a Data of 1 MLP child was missing.

b Data of 2 term-born children and 1 MLP child were missing.

* *p* < 0.05;

** *p* < 0.01;

*** *p* < 0.001.

See Table 3 for the correlations between the variables, per MLP group (upper diagonal half) and term-born group (lower diagonal half).

**Table 3:** Correlation table.

	1	2	3	4	5	6	7	8	9
1. Maternal NA	–	0.03	−0.11	−0.00	0.06	−0.11	0.32^*^	0.25	0.23
2. Maternal Stimulation	0.05	–	0.37^**^	−0.15	0.31^*^	0.19	−0.10	−0.09	0.11
3. Reciprocal engagement	0.00	0.39^**^	–	−0.63^***^	0.23	0.20	−0.26	−0.03	−0.11
4. Negative Interaction	−0.20	−0.20	−0.44^**^	–	−0.19	−0.15	0.09	0.01	0.11
5. Emotional Support	0.00	0.27	0.34^*^	−0.29	–	0.00	0.02	−0.10	0.10
6. Mother-led	−0.21	0.15	0.09	−0.03	−0.06	–	−0.15	−0.08	−0.10
7. Social–emotional difficulties	0.19	−0.03	−0.36^*^	0.12	−0.12	−0.20	–	0.35^**^	0.48^***^
8. Internalizing problem behavior	0.11	0.07	−0.14	0.10	−0.09	−0.29	0.40^**^	–	0.46^***^
9. Externalizing problem behavior	0.10	0.03	−0.05	0.13	−0.09	−0.27	0.30	0.64^***^	–

Pearson correlations are presented per MLP group (upper diagonal half) and term-born group (lower diagonal half).

* *p* < 0.05;

** *p* < 0.01;

*** *p* < 0.001.

### Moderated mediation model

**Figure 2:**
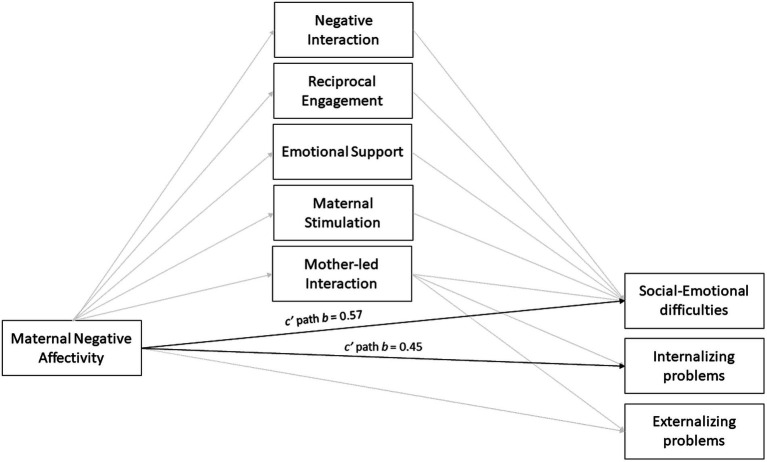
This figure shows the results of the three outcome models of the MLP sample (*n* = 108). Bold lines represent significant paths and display unstandardized coefficients.

**Figure 3:**
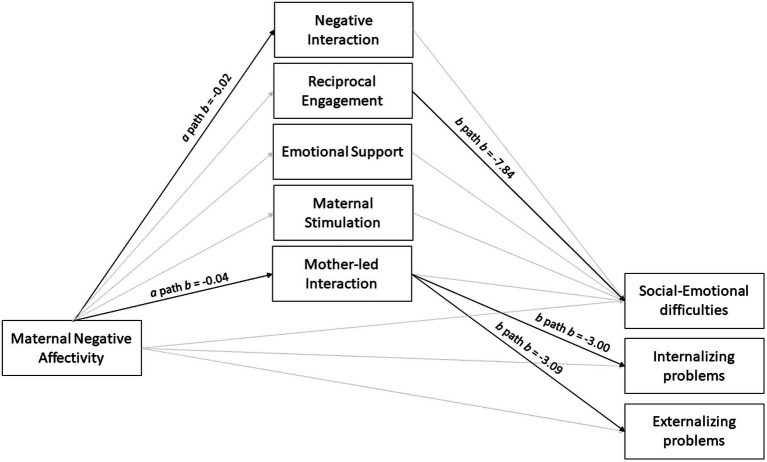
This figure shows the results of the three outcome models of the term-born children (*n* = 92). Bold lines represent significant paths and display unstandardized coefficients. The indirect effect through mother-led interaction (ab path) was significant for internalizing and externalizing problems.

Three models were run; one per psychosocial outcome measure. First, results are shown for maternal NA on mother–child interaction, representing only the a path (see Table 4). Table 5 shows the b, c’ and ab paths per outcome measure, including the index of moderated mediation effects. See also Figures 2, 3 for a visual representation of the results per MLP and term-born group, respectively.

**Table 4:** Mother–child interaction (mediator variables) as outcomes, representing the a paths of the moderated mediation model.

	Negative interaction			Reciprocal engagement			Maternal stimulation			Emotional support			Mother-led interaction		
	b (SE)	*t*	LL-UL	b (SE)	*t*	LL-UL	b (SE)	*t*	LL-UL	b (SE)	*t*	LL-UL	b (SE)	*t*	LL-UL
Constant	0.17 (0.25)	0.69	−0.31;0.66	0.07 (0.30)	0.22	−0.53;0.66	0.11 (0.32)	0.35	−0.52;0.74	−0.28 (0.26)	−1.08	−0.80;0.23	0.23 (0.41)	0.56	−0.57;1.03
NA	−0.02 (<0.01)^*^	−2.40	**−0.04;<−0.01**	<0.01 (0.02)	0.04	−0.03;0.03	<0.01 (0.02)	0.48	−0.03;0.04	<0.01 (<0.01)	0.18	−0.01;0.01	−0.04 (0.02)^*^	−2.23	**−0.07;<−0.01**
Birth status	0.02 (0.07)	0.26	−0.13;0.17	−0.16 (0.10)	−1.63	−0.36;0.03	−0.16 (0.12)	−1.34	−0.40;0.08	−0.02 (0.06)	−0.41	−0.13;0.09	−0.17 (0.13)	−1.33	−0.42;0.08
NA*birth status	0.02 (0.01)	1.42	−0.01;0.05	−0.01 (0.02)	−0.59	−0.05;0.03	<−0.01 (0.02)	−0.07	−0.05;0.05	0.01 (0.01)	0.58	−0.01;0.02	0.02 (0.02)	0.87	−0.03;0.07
Gender child	0.04 (0.07)	0.54	−0.12;0.19	−0.05 (0.10)	−0.50	−0.24;0.14	−0.05 (0.12)	−0.38	−0.28;0.19	0.01 (0.06)	0.17	−0.11;0.13	0.06 (0.12)	0.50	−0.18;0.31
Education medium	−0.15 (0.23)	−0.68	−0.62;0.30	−0.16 (0.29)	−0.55	−0.74;0.42	−0.09 (0.33)	−0.27	−0.73;0.56	0.28 (0.25)	1.14	−0.21;0.78	−0.07 (0.40)	−0.16	−0.86;0.73
Education high	−0.22 (0.23)	−0.99	−0.68;0.22	0.11 (0.29)	0.40	−0.45;0.68	0.02 (0.31)	0.07	−0.59;0.63	0.31 (0.25)	1.26	−0.18;0.80	−0.21 (0.39)	−0.53	−0.99;0.57
	Conditional effect of NA on negative interaction (a path)			Conditional effect of NA on reciprocal engagement (a path)			Conditional effect of NA on maternal stimulation (a path)			Conditional effect of NA on emotional support (a path)			Conditional effect of NA on Mother-led interaction (a path)		
MLP group	<−0.01 (0.01)	−0.28	−0.02;0.02	−0.01 (0.01)	−0.82	−0.04;0.02	0.01 (0.02)	0.38	−0.03;0.04	0.01 (0.01)	0.97	−0.01;0.02	−0.02 (0.02)	−1.08	−0.05; 0.02
Term-born group	−0.02 (0.01)^*^	−2.40	**−0.04;<−0.01**	<0.01 (0.02)	0.04	−0.03;0.03	0.01 (0.02)	0.48	−0.03;0.04	<0.01 (0.01)	0.18	−0.01;0.01	−0.04 (0.02)^*^	−2.23	**−0.07;<−0.01**
*R* ^2^	0.03			0.07			0.02			0.03			0.04		
*F*	1.98			2.06			0.58			0.44			1.44		

LL, lower limit for the 95% confidence intervals; UL, upper limit for the 95% confidence intervals; NA, negative affectivity; MLP, moderate to late preterm born children. Bold confidence intervals represent significant findings. Birth status is coded with 0 = term-born and 1 = moderate to late preterm born.

* *p* < 0.05;

** *p* < 0.01;

*** *p* < 0.001.

**Table 5:** Moderated-mediation model, representing the b, c’, and ab paths as well as the index of moderated mediation.

	Social–emotional difficulties^a^			Internalizing problems^b^			Externalizing problems^b^		
	b (SE)	t	LL-UL	b (SE)	t	LL-UL	b (SE)	t	LL-UL
Constant	25.24 (5.76)^***^	4.38	**13.88;36.60**	36.85 (4.22)^***^	8.72	**28.51;45.18**	47.49 (3.47)^***^	13.68	**40.64;54.34**
Negative affectivity	0.34 (0.34)	1.03	−0.32;1.01	−0.10 (0.21)	0.45	−0.33;0.52	0.11 (0.25)	0.43	−0.38;0.60
Negative interaction	−0.29 (3.04)	−0.10	−6.30;5.71	1.36 (2.03)	0.67	−2.63;5.36	2.65 (2.46)	1.07	−2.21;7.51
Reciprocal engagement	−7.84 (2.56)^**^	−3.07	**−12.88; −2.80**	−1.76 (1.59)	−1.11	−4.90;1.37	0.52 (1.93)	0.27	−3.29;4.33
Maternal stimulation	2.11 (1.72)	1.23	−1.20;5.51	2.17 (1.15)	1.88	−0.10;4.43	1.18 (1.28)	0.92	−1.35;3.71
Emotional support	−1.41 (2.97)	−0.47	−7.27;4.46	−2.16 (2.60)	−0.83	−7.29;2.98	−2.21 (2.57)	−0.86	−7.29;2.87
Mother-led interaction	−2.50 (1.38)	−1.82	−5.22;0.21	−3.00 (1.04)^**^	−2.89	**−5.04;–0.95**	−3.09 (1.15)^**^	−2.69	**−5.37;-0.82**
Birth status	−0.22 (1.74)	−0.13	−3.65;3.20	3.18 (1.26)^*^	−2.51	**0.69;5.67**	1.49 (1.33)	1.12	−1.13;4.11
NA*Birth status	0.23 (0.42)	0.54	−0.60-;1.05	0.35 (0.26)	1.35	−0.16;0.87	0.17 (0.30)	0.58	−0.41;0.76
Neg Int*Birth status	−1.52 (4.57)	−0.33	−10.53;7.50	−0.75 (3.46)	−0.22	−7.57;6.07	−1.57 (3.33)	−0.47	−8.15;5.01
Rec Eng*Birth status	3.63 (3.53)	1.03	−3.34;10.61	3.29 (2.42)	1.36	−1.49;8.07	−1.35 (2.51)	−0.54	−6.31;3.60
Maternal Stim*Birth status	−2.31 (2.39)	−0.97	−7.02;2.41	−2.91 (1.66)	−1.75	−6.19;0.37	0.34 (1.67)	0.20	−2.95;3.64
Emo Sup*Birth status	3.95 (5.50)	0.72	−6.90;14.80	−0.62 (3.49)	−0.18	−7.50;6.26	3.84 (3.33)	1.16	−2.72;10.40
Mother-led*Birth status	1.03 (1.98)	0.52	−2.88;4.93	2.46 (1.46)	1.69	−0.42;5.34	2.26 (1.47)	1.54	−0.63;5.16
Gender child	−1.30 (1.71)	−0.76	−4.67;2.07	0.63 (1.27)	0.50	−1.86;3.13	0.71 (1.26)	0.56	−1.77;3.19
Education medium	−4.69 (5.54)	−0.85	−15.62;6.25	5.92 (4.31)	1.38	−2.57;14.42	0.92 (3.20)	0.29	−5.39;7.23
Education high	−8.72 (5.37)	−1.62	−19.32;1.87	4.14 (4.15)	1.00	−4.05;12.33	−1.23 (3.23)	−0.38	−7.59;5.14
*R* ^2^	0.22^***^			0.16^**^			0.13		
*F*	3.24			2.56			1.69		
Conditional effect of Neg int on psychosocial outcome (b path)									
MLP group	−1.81 (3.37)	−0.54	−8.47;4.85	0.61 (2.81)	0.22	−4.93;6.16	1.08 (2.27)	0.47	−3.41;5.56
Term-born group	−0.29 (3.04)	−0.10	−6.30;5.71	1.36 (2.03)	0.67	−2.63;5.26	2.65 (2.46)	1.07	−2.21;7.51
Conditional effect of Rec Eng on psychosocial outcome (b path)									
MLP group	−4.21 (2.50)	−1.68	−9.15;0.73	1.53 (1.81)	0.84	−2.05;5.11	−0.84 (1.63)	−0.51	−4.05;2.38
Term-born group	−7.84 (2.56)^**^	−3.07	**−12.88;–2.80**	−1.76 (1.59)	−1.11	−4.90;1.37	0.52 (1.93)	0.27	−3.29;4.33
Conditional effect of Materernal Stim on psychosocial outcome (b path)									
MLP group	−0.19 (1.68)	−0.12	−3.50;3.12	−0.74 (1.91)	−0.62	−3.10;1.61	1.52 (1.08)	1.41	−0.60;3.65
Term-born group	2.11 (1.72)	1.23	−1.29;5.51	2.17 (1.15)	1.88	−0.11;4.43	1.18 (1.28)	0.92	−1.35;3.71
Conditional effect of Emo Sup on psychosocial outcome (b path)									
MLP group	2.54 (4.76)	0.53	−6.85;11.93	−2.78 (2.30)	−1.21	−7.32;1.76	1.63 (2.06)	0.79	−2.43;5.69
Term-born group	−1.41 (2.97)	−0.47	−7.28;4.46	−2.16 (2.60)	−0.83	−7.29;2.98	−2.21 (2.57)	−0.86	−7.29;2.87
Conditional effect of Mother-led on psychosocial outcome (b path)									
MLP group	−1.48 (1.40)	−1.05	−4.24;1.29	−0.53 (1.03)	−0.51	−2.57;1.50	−0.83 (0.93)	−0.90	−2.65;1.00
Term-born group	−2.50 (1.38)	−1.82	−5.22;0.21	−3.00 (1.04)^**^	−2.89	**−5.04;–0.95**	−3.09 (1.15)^**^	−2.69	**−5.37;–0.82**
Conditional direct effect NA on psychosocial outcome (c’ path)									
MLP group	0.57 (0.25)^*^	2.28	**0.08;1.06**	0.45 (0.15)^**^	2.91	**0.14;0.76**	0.28 (0.16)	1.72	−0.04;0.60
Term-born group	0.34 (0.34)	1.03	−0.32;1.01	0.10 (0.21)	0.45	−0.33;0.52	0.11 (0.25)	0.43	−0.38;0.60
Conditional indirect effect of negative interaction (ab path)									
MLP group	0.01 (0.03)		−0.05;0.08	<−0.01 (0.02)		−0.05;0.06	<−0.01 (0.02)		−0.05;0.06
Term-born	0.01 (0.06)		−0.11;0.14	−0.03 (0.04)		−0.13;0.04	−0.06 (0.06)		−0.19;0.03
Index of moderated mediation									
	<−0.01 (0.07)		−0.15;0.13	0.03 (0.05)		−0.06;0.14	0.05 (0.06)		−0.04;0.20
Conditional indirect effect of reciprocal engagement (ab path)									
MLP group	0.05 (0.07)		−0.06;0.22	−0.02 (0.04)		−0.10;0.05	0.01 (0.03)		−0.05;0.08
Term-born	−0.01 (0.12)		−0.28;0.22	<−0.01 (0.04)		−0.10;0.06	<0.01 (0.03)		−0.07;0.05
Index of moderated mediation									
	0.05 (0.14)		−0.20;0.37	−0.02 (0.05)		−0.11;0.10	0.01 (0.04)		−0.06;0.11
Conditional indirect effect of maternal stimulation (ab path)									
MLP group	<−0.01 (0.03)		−0.08;0.05	<−0.01 (0.02)		−0.05;0.04	0.01 (0.03)		−0.06;0.07
Term-born	0.02 (0.05)		−0.07;0.12	0.02 (0.04)		−0.07;0.10	0.01 (0.03)		−0.05;0.08
Index of moderated mediation									
	−0.02 (0.06)		−0.14;0.08	−0.02 (0.05)		−0.12;0.08	<−0.01 (0.04)		−0.10;0.08
Conditional indirect effect of emotional support (ab path)									
MLP group	0.02 (0.04)		−0.08;0.08	−0.02 (0.03)		−0.08;0.02	0.01 (0.02)		−0.03;0.06
Term-born	<−0.01 (0.02)		−0.05;0.03	<−0.01 (0.02)		−0.05;0.03	<−0.01 (0.02)		−0.04;0.04
Index of moderated mediation									
	−0.02 (0.05)		−0.08;0.10	−0.01 (0.03)		−0.08;0.05	0.01 (0.03)		−0.04;0.07
Conditional indirect effect of mother-led interaction (ab path)									
MLP group	0.02 (0.04)		−0.03;0.14	0.01 (0.03)		−0.04;0.07	0.02 (0.03)		−0.03;0.08
Term-born	0.10 (0.07)		−0.01;0.26	0.12 (0.06)		**0.02;0.26**	0.13 (0.07)		**0.01;0.29**
Index of moderated mediation									
	−0.07 (0.08)		−0.24;0.08	−0.11 (0.07)		−0.26;0.01	−0.11 (0.08)		−0.28;0.02

LL, lower limit for the 95% confidence intervals; UL, upper limit for the 95% confidence intervals. NA, negative Affectivity; Rec Eng, reciprocal engagement; Maternal Stim, maternal Stimulation; Emo Sup, emotional support; Motherled, mother-led interaction. Birth status is coded with 0 = term-born and 1 = moderate to late preterm born. Bootstrapped results are shown for the indirect effects. No *t* values are provided for bootstrapped results. Bold confidence intervals represent significant findings.

a Data of 1 MLP participant was missing, *n* = 199.

b Data of 2 term-born children and 1 MLP child were missing, *n* = 197.

* *p* < 0.05;

** *p* < 0.01;

*** *p* < 0.001.

#### Mother–child interaction

See Table 4 for all results on mother–child interaction. In the term-born group, maternal NA negatively predicted negative interaction (*b* = −0.02, *p* = 0.02) and mother-led interaction (*b* = −0.04, *p* = 0.03). In the MLP group these relationships were not found (*b*_negative interaction_ = <−0.01, *p* = 0.78; *b*_mother-led interaction_ = −0.02, *p* = 0.28). These relations did not significantly differ between the groups, as no significant moderation effect of birth status was found. Maternal NA did not predict emotional support, maternal stimulation and reciprocal engagement in both groups.

#### Social–emotional difficulties

See Table 5 for the results on social–emotional difficulties. The total model explained 22% of the variance in social–emotional difficulties (*p* < 0.001). The results showed a significant positive, direct effect of maternal NA on social–emotional difficulties for the MLP group (*b* = 0.57, *p* = 0.02). A non-significant positive effect was seen for the term-born group (*b* = 0.34, *p* = 0.31). These relations were not significantly different between the groups, as no moderation effect of birth status was found (*p* = 0.59). Reciprocal engagement negatively predicted social–emotional difficulties in term-born children (*b* = −7.84, *p* = 0.003), but not in MLP children (*b* = −4.21, *p* = 0.09). No moderation effect of birth status was found for this relation (*p* = 0.31), indicating that the relationship between reciprocal engagement and social–emotional difficulties was not significantly different between the groups. No significant relationships were found with the four remaining mother–child interaction variables.

#### Internalizing problem behavior

See Table 5 for the results on internalizing problems. The total model explained 16% of the variance in internalizing problems (*p* = 0.001). The results showed a direct, positive effect for maternal NA on internalizing problems in the MLP group (*b* = 0.45, *p* = 0.004). This relation was not found in term-born children (*b* = 0.10, *p* = 0.65). The relationships were not significantly different between groups (i.e., no moderation of birth status, *p* = 0.18). For term-born children, a mediation effect was found through mother-led interaction (ab path, *b* = 0.12, 95%BootCI = 0.02 to 0.26), in which maternal NA predicted lower mother-led interaction (a path, *b* = −0.04, *p* = 0.02) which in turn predicted more internalizing problems (b path, *b* = −3.00, *p* = 0.004). This mediation effect was not found in the MLP group. No moderated mediation effects were found of birth status on this relation (*b* = −0.11, 95%BootCI = −0.28 to 0.02). No significant relationships were found with the four remaining mother–child interaction variables.

#### Externalizing problems

See Table 5 for the results on externalizing problems. The total model explained 13% of the variance in externalizing problems (*p* = 0.05). The results showed no direct effect for maternal NA on externalizing problems for both groups (MLP: *b* = 0.28, *p* = 0.09, term-born: *b* = 0.11, *p* = 0.67). For the term-born group, a mediation effect was found through mother-led interaction (ab path, *b* = 0.13, 95%BootCI = 0.01 to 0.29) in which higher levels of maternal NA predicted less mother-led interaction (a path, *b* = −0.04, *p* = 0.02), which in turn predicted more externalizing problems (b path, *b* = −3.09, *p* = 0.008). This mediation effect was not found in the MLP group. No significant moderated mediation effects were found of birth status for this relationship (*b* = −0.11, 95% BootCI = −0.26 to 0.01). No significant relationships were found with the four remaining mother–child interaction variables.

## Discussion

The current study examined whether maternal NA was related to psychosocial difficulties in MLP and term-born toddlers, and if this was mediated by quality of mother–child interaction. Additionally, it was studied if these relationships were different for MLP children compared to term-born children (i.e., moderation effect of birth status). Our results showed that mothers with higher levels of maternal NA, which is reflected by the tendency to experience negative thoughts, feelings and emotions, reported more psychosocial difficulties in their toddlers. For MLP children, a direct relationship between maternal NA and psychosocial outcomes was found, which was not mediated by mother–child interaction. For term-born children, maternal NA was indirectly associated with psychosocial child outcomes, through levels of mother-led interaction. Birth status did not moderate these relationships, indicating that the associations found within the groups were not clearly different from each other. However, the within group findings are discussed here as well, as these may be important for future studies.

In term-born children, mother-led interaction – one of the five observed mother–child interactions–formed a mediating factor. Higher levels of maternal NA were related to lower levels of mother-led interaction, which subsequently predicted more internalizing and externalizing problems in term-born children. This is in line with previous research showing that maternal depressive symptoms are related to more passive and withdrawn maternal behaviors (Stein et al., 2012; Esposito et al., 2017) – i.e. lower levels of mother-led interaction – which negatively affect children’s psychosocial functioning (Easterbrooks et al., 2012).

In addition to this mediation effect of mother-led interaction, we found non-mediating associations of mother–child interaction within the group of term-born children. Higher levels of reciprocal engagement were related to less social–emotional difficulties of the children, which also is in line with previous research (Feldman et al., 2013). Surprisingly, higher levels of maternal NA were linked to lower levels of negative interaction within the term-born group, whereas we expected to find the opposite. To interpret this finding, it is important to keep in mind that in the current study negative interaction was mainly based on the child’s behavior (e.g., negative emotionality or labile affect). It might be that term-born children respond adaptively towards higher levels of maternal NA by avoiding tension and problems, resulting in lower scores on negative interaction. Future studies are needed to confirm these hypotheses.

In MLP children, maternal NA was directly related to psychosocial problems. Contrary to our expectations, no mediation effects nor associations were found for mother–child interaction, indicating that mother–child interaction was not related to maternal NA and psychosocial outcomes for MLP toddlers. The absence of these associations could be due to lower neurodevelopmental functioning of MLP children compared to term-born children, which may have hindered their potential to develop through mother–child interaction. Previous research found that preterm children showed more withdrawn behavior, lower self-regulation skills, less alertness and less clear cues for the mother to interpret (White-Traut et al., 2002; Feldman and Eidelman, 2006; Pickler et al., 2010; Moe et al., 2016). We also found that the MLP group showed more internalizing problems – which includes withdrawn behaviors – and scored lower on reciprocal engagement – which includes aspects of attentional and regulation skills such as joint attention and on task persistence. Moreover, previous research on the current sample showed that the MLP group indeed had lower neurodevelopmental outcomes, as shown by lower scores on cognitive-, motor- and language/communication skills than term-born children at 24 months of age. After correcting age for prematurity, still a delay in receptive communication skills was found (De Jong et al., 2015). Difficulties in receptive communication skills can hinder the child in understanding the parent’s communication. This can make it more challenging for the MLP child to engage in the interaction – which is in line with our finding of lower scores on reciprocal engagement in the MLP group – and benefit from it. We speculate that MLP children may be somewhat less active, focused and engaged during interactions. This might complicate the opportunities to learn and develop through mother–child interaction. We hypothesize that MLP children need more guidance and active behaviors from the mother than was found in the interaction observed for the current study, in order to be engaged in the interaction and to have them benefit in terms of their psychosocial development. Future studies could investigate whether an increase in active and leading behaviors of the mother would evoke more active behaviors of preterm born children.

Another explanation for the non-significant relations between mother–child interaction and the other studied variables in the MLP group could be due to our operationalization of the interaction. A 10 minutes structured task consisting of reading a book and making a puzzle together may not have given a complete representation of all (subtle) mother–child characteristics. It could be that different and perhaps more subtle mechanisms play a role in MLP mother–child dyads. Interestingly, only reciprocal engagement was lower in the MLP group compared to the term-born group, but the remaining four mother–child interaction characteristics were not of different quality. Future research is needed to investigate mother–child interaction in greater detail. We suggest an approach in which consistency across patterns can be observed and a wider variety of characteristics of mother–child interactions are elicited. This could be reached by including a greater variety of tasks than reading a book and making a puzzle, and observing for multiple days in a row or over a longer period of time, so that subtle patterns within the mother–child dyad may become clearer.

When interpreting the findings of the current study, it is important to keep in mind that – although our results suggest different patterns of relations in term-born versus MLP children–the differences in these patterns (e.g., moderation effects) did not reach statistical significance. Therefore, more research focusing on moderated-mediation analyses using larger sample sizes is advised to confirm if such different patterns exist. Other limitations of our study are that we assessed maternal NA once and are therefore unaware of the stability of this trait throughout the study. However, previous research showed that the outcomes of the DS14, which was used to assess NA, are relatively stable over time (Kupper et al., 2013). Nonetheless, for future research it is advised to measure maternal NA at multiple time points throughout the study as some variation in scores over time may occur. Another limitation is that mothers filled out the questionnaires for maternal NA as well as for psychosocial difficulties of their child, which could have elicited a response bias. However, we found no significant correlation between maternal NA and psychosocial outcomes for the term-born group, indicating that a response bias is unlikely. Lastly, the internal consistency of the ASQ-SE for the current sample was relatively low (i.e., α = 0.45), indicating that not one single underlying construct has been measured. This is not surprising due to the broad domain of social–emotional development that the ASQ-SE measures, but it is important to keep in mind that the current results of the ASQ-SE give information about the *overall* social–emotional difficulties. Further research using instruments targeting specific dimensions of social–emotional development may provide more insight into which dimensions are most affected by maternal NA and/or mother–child interaction.

Despite its limitations, the current study contains several strenghts. In addition to its prospective longitudinal design, the fact that both self-report and observational measures were used decreases the chance of response bias. Furthermore, the preterm group consisted of relatively low-risk MLP children, a group that is studied less often than extreme and very preterm children, though MLP children form a large proportion of all children born preterm. Our results indicated that this group was not at a very high risk for problems, as we found that externalizing problems and social–emotional difficulties were comparable to the term-born group, just as four out of the five observed characteristics of mother–child interactions. Nevertheless, internalizing problems were higher and reciprocal engagement was lower in the MLP children.

For clinical practice it is advised to pay attention to levels of NA in mothers, regardless of birth status of the child. Mothers scoring high on NA could be offered additional support. Furthermore, for mothers of term-born children, the focus could be directed towards quality of mother–child interaction–specifically in stimulating mothers to show leading, active and engaged behaviors as these seem to be predictive of the term-born child’s psychosocial outcomes. For MLP children, future research should clarify whether increasing levels of leading and active behaviors of the mother is beneficial for MLP children.

To conclude, higher levels of maternal NA are associated with more psychosocial problems in toddlers, directly for MLP children and indirectly for term-born children through levels of mother-led interaction.

## Data availability statement

The data analyzed in this study is subject to the following licenses/restrictions: The data analyzed in this study can be shared upon reasonable request. Requests to access these datasets should be directed to LK, l.j.g.krijnen@uu.nl.

## Ethics statement

The studies involving human participants were reviewed and approved by Utrecht Medical Center Ethics Committee, identification code NL34143.041.10. Written informed consent to participate in this study was provided by the participants’ legal guardian/next of kin.

## Author contributions

LK: conceptualization, methodology, formal analysis, and writing—original draft. MV: conceptualization, writing—review and editing, and supervision. AB: conceptualization, writing—review and editing, supervision. All authors contributed to the article and approved the submitted version.

## Conflict of interest

The authors declare that the research was conducted in the absence of any commercial or financial relationships that could be construed as a potential conflict of interest.

## Publisher’s note

All claims expressed in this article are solely those of the authors and do not necessarily represent those of their affiliated organizations, or those of the publisher, the editors and the reviewers. Any product that may be evaluated in this article, or claim that may be made by its manufacturer, is not guaranteed or endorsed by the publisher.
